# Mechanical circulatory support for refractory out-of-hospital cardiac arrest: a Danish nationwide multicenter study

**DOI:** 10.1186/s13054-021-03606-5

**Published:** 2021-05-22

**Authors:** Sivagowry Rasalingam Mørk, Carsten Stengaard, Louise Linde, Jacob Eifer Møller, Lisette Okkels Jensen, Henrik Schmidt, Lars Peter Riber, Jo Bønding Andreasen, Sisse Anette Thomassen, Helle Laugesen, Phillip Michael Freeman, Steffen Christensen, Jacob Raben Greisen, Mariann Tang, Peter Hasse Møller-Sørensen, Lene Holmvang, Emilie Gregers, Jesper Kjaergaard, Christian Hassager, Hans Eiskjær, Christian Juhl Terkelsen

**Affiliations:** 1 Department of Cardiology, Aarhus University Hospital, Palle Juul-Jensens Boulevard 99, 8200 Aarhus N, Denmark; 2 Department of Cardiology, Odense University Hospital, Odense, Denmark; 3 Department of Anaesthesiology and Intensive Care, Odense University Hospital, Odense, Denmark; 4 Department of Thoracic and Vascular Surgery, Odense University Hospital, Odense, Denmark; 5 Department of Anaesthesiology and Intensive Care, Aalborg University Hospital, Aalborg, Denmark; 6 Department of Cardiology, Aalborg University Hospital, Aalborg, Denmark; 7 Department of Anaesthesiology and Intensive Care, Aarhus University Hospital, Aarhus, Denmark; 8 Department of Thoracic and Vascular Surgery, Aarhus University Hospital, Aarhus, Denmark; 9 Cardiothorascic Anaesthesiology, Copenhagen University Hospital, Copenhagen, Denmark; 10 Department of Cardiology, Copenhagen University Hospital, Copenhagen, Denmark; 11 The Danish Heart Foundation, Copenhagen, Denmark

**Keywords:** Out-of-hospital cardiac arrest, Mechanical circulatory support, Extracorporeal membrane oxygenation, Impella, Cardiopulmonary resuscitation

## Abstract

**Background:**

Mechanical circulatory support (MCS) with either extracorporeal membrane oxygenation or Impella has shown potential as a salvage therapy for patients with refractory out-of-hospital cardiac arrest (OHCA). The objective of this study was to describe the gradual implementation, survival and adherence to the national consensus with respect to use of MCS for OHCA in Denmark, and to identify factors associated with outcome.

**Methods:**

This retrospective, observational cohort study included patients receiving MCS for OHCA at all tertiary cardiac arrest centers (n = 4) in Denmark between July 2011 and December 2020. Logistic regression and Kaplan–Meier survival analysis were used to determine association with outcome. Outcome was presented as survival to hospital discharge with good neurological outcome, 30-day survival and predictors of 30-day mortality.

**Results:**

A total of 259 patients were included in the study. Thirty-day survival was 26%. Sixty-five (25%) survived to hospital discharge and a good neurological outcome (Glasgow–Pittsburgh Cerebral Performance Categories 1–2) was observed in 94% of these patients***.*** Strict adherence to the national consensus showed a 30-day survival rate of 30% compared with 22% in patients violating one or more criteria. Adding criteria to the national consensus such as signs of life during cardiopulmonary resuscitation (CPR), pre-hospital low-flow < 100 min, pH > 6.8 and lactate < 15 mmol/L increased the survival rate to 48%, but would exclude 58% of the survivors from the current cohort. Logistic regression identified asystole (RR 1.36, 95% CI 1.18–1.57), pulseless electrical activity (RR 1.20, 95% CI 1.03–1.41), initial pH < 6.8 (RR 1.28, 95% CI 1.12–1.46) and lactate levels > 15 mmol/L (RR 1.16, 95% CI 1.16–1.53) as factors associated with increased risk of 30-day mortality. Patients presenting signs of life during CPR had reduced risk of 30-day mortality (RR 0.63, 95% CI 0.52–0.76).

**Conclusions:**

A high survival rate with a good neurological outcome was observed in this Danish population of patients treated with MCS for OHCA. Stringent patient selection for MCS may produce higher survival rates but potentially withholds life-saving treatment in a significant proportion of survivors.

**Supplementary Information:**

The online version contains supplementary material available at 10.1186/s13054-021-03606-5.

## Background

Out-of-hospital cardiac arrest (OHCA) is a time-critical condition associated with a high mortality worldwide. Despite various initiatives to improve public engagement and ensure access to a sufficient number of external defibrillators, survival rates remain poor [1]. Short-term mechanical circulatory support (MCS) with extracorporeal membrane oxygenation (ECMO) or Impella devices has emerged as a rescue therapy in adult patients with OHCA that is refractory to conventional cardiopulmonary resuscitation (CPR). MCS may ensure life-saving organ perfusion, lending clinicians crucial time to identify and treat the underlying cause of cardiac arrest. Extracorporeal cardiopulmonary resuscitation (ECPR) refers to the rapid application of ECMO in the setting of refractory cardiac arrest. Several observational studies and recently one randomized clinical trial have demonstrated encouraging results after ECPR [2–6]. In recent years, the use of Impella devices as MCS for refractory OHCA have also shown potential in this high-risk population [7, 8]. Although the field of MCS for refractory cardiac arrest has evolved rapidly over the past decades, identifying optimal candidates is still an inevitable challenge.

MCS for refractory OHCA has been introduced gradually over the past ten years in Denmark, and a national consensus was adopted in February 2018 [9]. However, detailed knowledge of the full cohort treated remains scarce. Regional differences in triage of patients with OHCA may influence the availability of MCS, which may in turn affect patient selection and outcome. The aims of this study were to describe temporal trends and regional variation in the use of MCS in Denmark, to evaluate adherence to the national consensus on ECPR use and to identify factors associated with outcome.

## Methods

This nationwide retrospective, observational cohort study was conducted at four tertiary cardiac arrest centers in Denmark (Aalborg University Hospital, Aarhus University Hospital, Odense University Hospital and Copenhagen University Hospital). In Denmark, MCS for refractory OHCA is performed at these four centers exclusively.

### National consensus

**Fig. 1:**
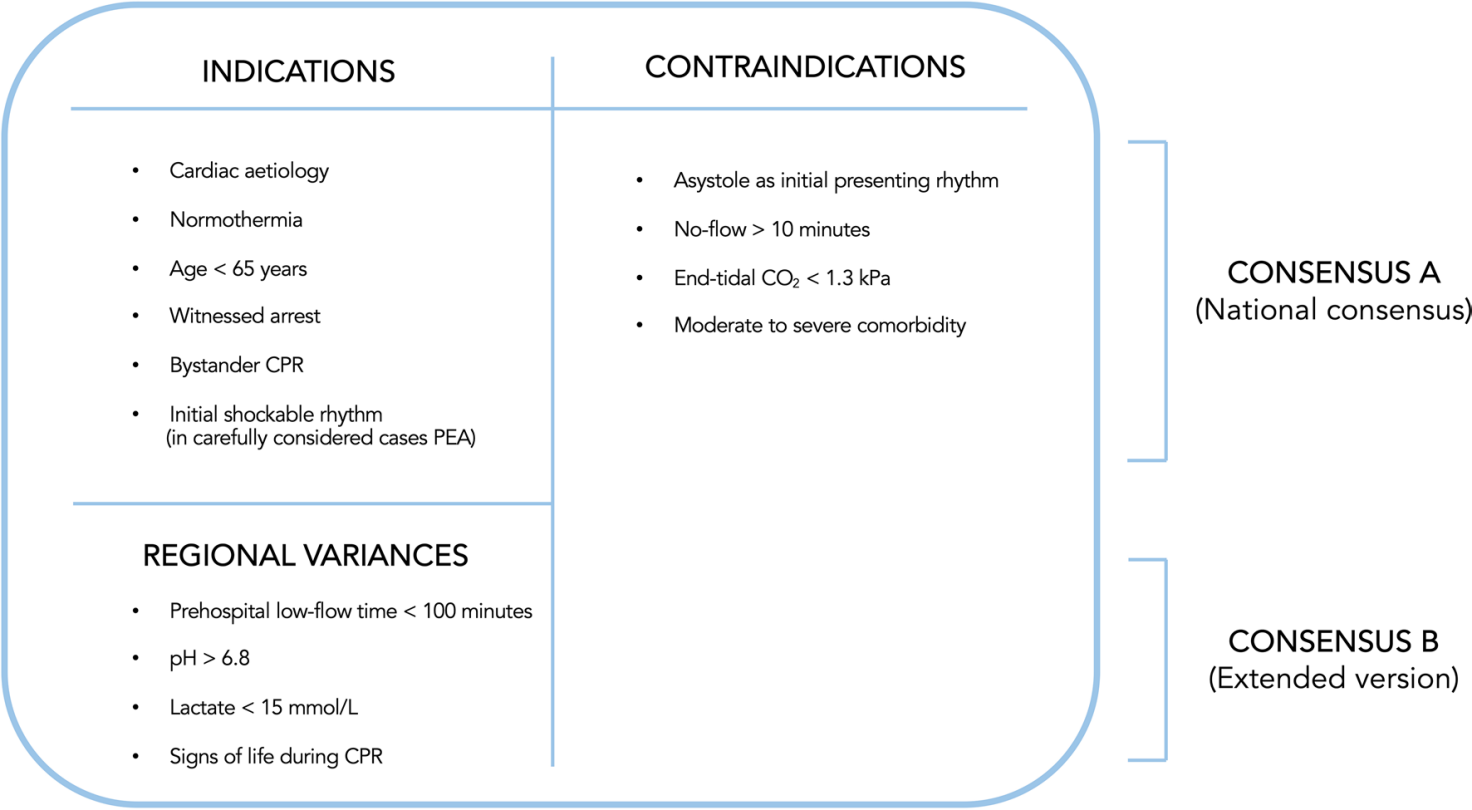
National consensus and extended consensus with regional variances for selection of patients with refractory OHCA and possible candidates for mechanical circulatory support

The Danish national consensus on the use of ECPR in patients with refractory OHCA was adopted in February 2018 [9] (Fig. 1, Consensus A—National consensus). The most consistent criteria for inclusion were normothermic cardiac arrest with a presumed cardiac origin, age < 65 years, an initial shockable rhythm, witnessed arrest, bystander CPR and end-tidal CO_2_ > 1.3 kPa. Regional differences in the inclusion criteria were present during the study period as shown in Fig. 1, Consensus B—Extended version and Additional file 1: Table S1.

### Study population

The study population included all patients aged ≥ 18 years receiving MCS for refractory OHCA, which was defined as absence of return of spontaneous circulation (ROSC) despite resuscitation efforts for more than 15 min. Patients treated between July 2011 and December 2020 were identified from local MCS databases and medical records. Due to regional differences in MCS availability and updates to databases, the data collection period differed for each hospital: Aalborg University Hospital (February 2016–December 2020), Aarhus University Hospital (July 2011–December 2020), Odense University Hospital (November 2015–December 2020) and Copenhagen University Hospital (November 2016–December 2020).

### Study setting

Patient selection, triage, and implementation of either Impella or ECPR were performed at the discretion of the treating ECPR team at the individual centers. The specialized ECPR teams, including anaesthesiologists, cardiothoracic surgeons, perfusionists, and invasive and general cardiologists managed all patients upon arrival at the catherization laboratory. Venoarterial ECMO cannulations were inserted percutaneously using the Seldinger technique with ultrasound guidance. In case of unsuccessful percutaneous cannulation, open cut-down technique with direct visualization of the femoral vein and artery was performed. Vascular access was achieved by 15F-23F arterial cannulas and 19F-26F venous cannulas. In the majority of patients, a distal arterial perfusion cannula was inserted to ensure antegrade limb perfusion. Circuit flow was titrated until effective circulatory response was achieved. Fluid, inotropes and vasopressors were applied if necessary. ECPR was achieved in most cases; however, in a minority of patients, the Impella device was initially employed if visible spontaneous cardiac contractility was observed on echocardiography during rhythm check. The Impella device was placed percutaneously in the femoral artery, and correct positioning was then confirmed by fluoroscopy or echocardiography. ECPR in combination with Impella was applied in patients with failing cardiac recovery immediately after ECPR initiation or within 24 h after. Combined use of ECPR and Impella was initiated to ensure aortic valve opening and sufficient left ventricular unloading.

For both ECPR- and Impella-treated patients, unfractionated heparin was administrated routinely to avoid systemic clotting and aiming for an activated partial thromboplastin time of 60–80 s or an activated clotting time of 160–180 s.

Post-resuscitation care and management were performed according to local intensive care unit (ICU) standard protocols including targeted temperature management (TTM), neurological prognostication and procedures for withdrawal of treatment. Weaning from MCS was done if cardiac and respiratory function were considered to have recovered sufficiently or if further treatment was deemed futile [10].

### Data collection

Study data were recorded in a uniform national database. A study coordinator from each hospital was assigned to manage the data collection. According to the Utstein recommendation for data collection [11], information on cardiac arrest was acquired from the pre-hospital emergency medical service logistic systems and included information on: time of cardiac arrest, witnessed arrest, bystander CPR, initial rhythm and pre-hospital care comprising inotropic usage and intubation. Patient demographics and in-hospital data on clinical parameters, known comorbidities, laboratory tests, intervention and outcome data were obtained from patient records.

### Study end-points

The primary end-point was 30-day survival. Secondary end-points included survival to hospital discharge, neurological outcome at hospital discharge and regional differences in triage and outcome. Neurological outcome was evaluated by the Glasgow–Pittsburgh Cerebral Performance Categories (CPC), and a favorable outcome was defined as CPC scores 1 and 2 [12].

### Statistical analysis

Continuous data are presented as median and interquartile range (IQR, P_25_-P_75_) and categorical data as number and percentages. The Mann–Whitney U test and the Kruskal–Wallis H test were used for comparison of continuous data, whereas the chi-squared test and Fisher’s exact test were used for categorical data. Logistic regression was performed to assess the association of risk factors on 30-day mortality. Results are expressed as risk ratio (RR) and 95% confidence interval (CI). Risk factors were identified a priori based on their clinical relevance and previously published literature. Survival analysis results are presented as Kaplan–Meier curves for various subgroups of patients and compared with the log rank test. In case of missing values, patients were excluded from the statistical analysis. Two-sided p-values of < 0.05 were considered statistically significant. Statistical tests were performed using STATA/IC 16, College Station TX77845, USA, for Mac.

## Results

Additional file 2: Figure S1 demonstrates the gradual implementation of MCS in Denmark. Between July 2011 and December 2020, a total of 259 patients treated with MCS for OHCA were enrolled in the study: (Aalborg University Hospital, n = 34, Aarhus University Hospital, n = 138, Odense University Hospital, n = 55, and Copenhagen University Hospital, n = 32). Survival to day 30 was seen in 67 (26%). A total of 65 (25%) patients survived to hospital discharge, and 61 (94%) of these patients were discharged with a CPC of 1–2.

### Baseline and cardiac arrest characteristics

Baseline and arrest characteristics are summarized in Table 1. The median age of the study population was 53 years (IQR, 45–60 years), and 79% were men. No significant differences in comorbidities were seen between survivors and non-survivors. In most cases, arrest aetiology was of cardiac origin; and the three dominant causes were acute myocardial infarction (n = 142, 55%), primary arrhythmia (n = 42, 16%) and pulmonary embolism (n = 24, 9%). Significantly more survivors than non-survivors presented an initial shockable rhythm (p = 0.002) and signs of life during conventional CPR (p < 0.001). Witnessed cardiac arrest was present in 223 (86%) of the patients, and 246 (95%) patients received bystander CPR initiated immediately after recognition of arrest with a median no-flow time of 0 min (IQR, 0–1 min). Survivors experienced a significantly shorter total low-flow time from cardiac arrest to MCS initiation than non-survivors (94 min versus 107 min, p = 0.002).

**Table 1:** Baseline characteristics and pre-hospital data stratified by 30-day survival status

Variable	Total (n = 259)	Survivors (n = 67)	Non-survivors (n = 192)	*P-value*
Age (years)	53 [45–60]	54 [46–62]	53 [43–59]	0.19
Age ≤ 65 years	221 (85)	56 (84)	165 (86)	0.64
Male sex	205 (79)	50 (75)	155 (81)	0.29
*Comorbidities*				
History of ischemic heart disease	30 (12)	9 (13)	21 (11)	0.58
Previous myocardial infarction	28 (11)	8 (12)	20 (11)	0.75
History of congestive heart disease	19 (7)	4 (6)	15 (8)	0.62
Hypertension	65 (25)	18 (27)	47 (25)	0.73
Type 2 diabetes	26 (10)	3 (5)	23 (12)	0.06
Peripheral vascular disease	11 (4)	4 (6)	7 (4)	0.31
Previous chronic kidney disease	8 (3)	2 (3)	6 (3)	0.65
Previous stroke	10 (4)	0 (0)	10 (5)	0.05
*Cause of cardiac arrest*				
Acute myocardial infarction	142 (55)	42 (63)	100 (52)	0.13
Pulmonary embolism	24 (9)	8 (12)	16 (8)	0.26
Primary arrhythmia	42 (16)	10 (15)	32 (17)	0.85
Chronic heart disease	5 (2)	1 (2)	4 (2)	1.00
Cerebral	6 (2)	0 (0)	6 (3)	0.34
Toxic	10 (4)	3 (5)	7 (4)	0.72
Other	20 (8)	4 (6)	16 (8)	0.79
Unknown	13 (4)	0 (0)	13 (5)	0.02
Witnessed arrest	223 (86)	60 (90)	163 (85)	0.34
Bystander CPR	246 (95)	64 (96)	182 (95)	0.94
Transient ROSC	48 (19)	27 (40)	21 (11)	< 0.001
Signs of life during CPR	100 (39)	45 (67)	55 (29)	< 0.001
*Initial presenting rhythm*				
Shockable (VT/VF)	173 (67)	55 (82)	118 (62)	0.002
PEA	57 (22)	10 (15)	47 (25)	0.10
Asystole	28 (11)	2 (3)	26 (14)	0.02
End-tidal CO_2_	3.6 [2.8–5.0]	3.6 [2.9–5.0]	3.5 [2.5–5.0]	0.99
Mechanical compression (LUCAS)	235 (91)	61 (91)	174 (91)	0.97
No-flow (min)	0 [0–1]	0 [0–2]	0 [0–1]	0.77
Pre-hospital low-flow (min)	72 [58–90]	67 [46–90]	75 [60–90]	0.06
Total low-flow (min)	105 [86–125]	94 [73–120]	107 [90–127]	0.002

Abbreviations: *CPR* Cardiopulmonary resuscitation; *ROSC* Return of spontaneous circulation; *VT* Ventricular tachycardia; *VF* Ventricular fibrillation*; PEA* Pulseless electrical activity; *LUCAS* Lund University cardiopulmonary assist system

Values are stated as medians and interquartile range [IQR] or numbers and percentages. A p value < 0.05 is considered significant

### In-hospital and outcome characteristics

In-hospital and outcome data are shown in Table 2. ECPR was established in 225 (86.9%) patients, whereas Impella assistance was commenced in 12 (4.6%) patients. Twenty-two patients (8.5%) received concomitant support (ECPR + Impella). Among the 247 patients treated with ECPR, cannulation was done percutaneously in 224 patients and by cut-down in 23 patients. Distal perfusion was established in 179 of the ECPR patients. Acute coronary angiography was performed in 234 (90%) patients, and 124 (48%) of the patients received percutaneous coronary intervention. Survivors more likely presented advantageous blood gas analysis with higher median pH levels (7.01 versus 6.88, p < 0.001) and lower serum lactate levels (12.0 mmol/L versus 15.0 mmol/L, p < 0.001) prior to MCS implantation. The majority of the patients (n = 218; 84%) were admitted directly to the ICU after MCS commencement. However, in 41 (16%) patients, further resuscitation efforts were deemed futile and treatment was withdrawn before admission to the ICU. The main reasons for terminating treatment were anoxic brain injury after cardiac arrest or intracerebral haemorrhage, extensive bleeding due to either acute aortic dissection or complication of Lund University Cardiopulmonary Assist System (LUCAS) with spleen or liver rupture, and severe heart failure despite inotropes and vasopressor support.

**Table 2:** In-hospital data stratified by 30-day survival status

Variable	Total (n = 259)	Survivors (n = 67)	Non-survivors (n = 192)	*P-value*
ECMO (only)	225 (86.9)	55 (82)	170 (89)	0.18
Impella (only)	12 (4.6)	6 (9)	6 (3)	0.05
ECMO + Impella	22 (8.5)	6 (9)	16 (8)	0.88
*Laboratory data upon arrival*				
pH	6.90 [6.82–7.02]	7.01 [6.92–7.15]	6.88 [6.80–6.98]	< 0.001
Lactate (mmol/L)	14.4 [11.4–17.0]	12.0 [9.1–14.7]	15.0 [12.0–19.0]	< 0.001
Potassium (mmol/L)	4.4 [3.7–5.4]	4.1 [3.6–4.9]	4.6 [3.8–5.5]	0.06
Hemoglobin (mmol/L)	8.6 [7.5–9.6]	8.7 [8.1–9.6]	8.6 [7.3–9.6]	0.22
Creatinine (mmol/L)	114 [98–130]	109 [95–131]	115 [99–130]	0.86
CAG performed	234 (90)	62 (93)	172 (90)	0.48
Coronary intervention (PCI/stent)	124 (48)	37 (55)	87 (46)	0.20
Left main	22 (9)	5 (8)	17 (10)	0.73
Left anterior descending	76 (33)	27 (44)	49 (29)	0.02
Left circumflex	8 (3)	2 (3)	6 (4)	0.96
Right coronary artery	30 (13)	9 (15)	21 (12)	0.58
*Intensive care stay*				
No. of patients admitted to ICU	218 (84)	67 (100)	151 (79)	< 0.001
TTM	152 (70)	46 (69)	106 (70)	0.46
Renal replacement therapy	91 (42)	32 (48)	59 (31)	0.27
ICU length of stay (hours)	53 [13–238]	284 [163–528]	19 [10–63]	< 0.001
*ECPR-/Impella-related complications*				
Bleeding at cannulation site	76 (29)	32 (48)	44 (23)	0.01
Limb ischemia	24 (9)	5 (8)	19 (10)	0.31
Gastrointestinal bleeding	33 (13)	10 (15)	23 (12)	0.90
Gastrointestinal ischemia	22 (9)	4 (6)	18 (9)	0.20
Time on ECPR (hours)	50 [27–95]	67 [40–98]	37 [8–77]	0.002
Time on Impella (hours)	74 [28–165]	62 [50–165]	84 [10–166]	0.46
Hospital length of stay (hours)	23 [7–358]	687 [496–1060]	13 [4–46]	< 0.001

Abbreviations: *ECMO* Extracorporeal membrane oxygenation; *CAG* Coronary angiogram; *PCI* Primary coronary intervention; *ICU* Intensive care unit; *TTM* Target temperature management

Values are stated as medians and interquartile range (IQR) or numbers and percentages. A p- value of < 0.05 is considered significant

No differences between the groups were seen regarding renal replacement therapy or TTM, (p = 0.27 and p = 0.46, respectively). MCS duration and ICU length of stay were longer in survivors, indicating rapid withdrawal of support in the case of treatment futility. Bleeding at the cannulation site was observed in 76 (29%) and limb ischemia was seen in 24 (9%) of the patients. None of the patients without distal perfusion had limb ischemia. The main cause of withdrawal of life-sustaining treatment in non-survivors was severe brain injury (n = 88, 46%), no cardiac recovery (n = 25, 13%), device failure (n = 4, 2%), multiorgan failure (n = 49, 26%) and other (n = 26, 14%).

### Predictors of 30-day mortality and survival outcomes

Table 3 shows results from the binary logistic regression. Thirty-day mortality was significantly associated with initial presenting rhythm with asystole (RR 1.36, 95% CI 1.18–1.57, p < 0.001), pulseless electrical activity (PEA) (RR 1.20, 95% CI 1.03–1.41, p = 0.02), low pH levels < 6.8 (RR 1.28, 95% CI 1.12–1.46, p < 0.001) and high lactate levels > 15 mmol/L (RR 1.33, 95% CI 1.16–1.53, p < 0.001). Signs of life during CPR (RR 0.63, 95% CI 0.52–0.76, p < 0.001) and transient ROSC (RR 0.54, 95% CI 0.39–0.76, p < 0.001) were both associated with a lower risk of mortality.

**Table 3:** Binary logistic regression analysis of risk factors associated with 30-day mortality

Variables	Number of valid cases	Univariate analysis		
	Total (n = 259)	RR	95% CI	*P-value*
Age (years)	259	1.00	(0.99–1.00)	0.21
Male sex	259	1.10	(0.90–1.34)	0.34
Witnessed arrest	259	0.91	(0.76–1.08)	0.28
Bystander CPR	259	0.98	(0.70–1.38)	0.93
Initial presenting rhythm	258			
VT/VF*		1.00	–	–
PEA		1.20	(1.03–1.41)	0.02
Asystole		1.36	(1.18–1.57)	< 0.001
Signs of life during CPR	251	0.63	(0.52–0.76)	< 0.001
Transient ROSC	252	0.54	(0.39–0.76)	< 0.001
End-tidal CO_2_	224	0.82	(0.65–1.01)	0.07
Pre-hospital low-flow ≤ 60 min	259	0.80	(0.67–0.95)	0.02
pH ≤ 6.8	236	1.28	(1.12–1.46)	< 0.001
Lactate ≥ 15 mmol/L	251	1.33	(1.16–1.53)	< 0.001

Abbreviations: *CPR* Cardiopulmonary resuscitation; *VT* Ventricular tachycardia; *VF* Ventricular fibrillation; *PEA* Pulseless electrical activity; *ROSC* Return of spontaneous circulation; *RR* Risk ratio; *CI* Confidence interval

*Reference group

Kaplan–Meier curves and the log rank test demonstrated similar results (Additional file 3: Figure S2). Patients presenting signs of life during CPR had a higher survival rate compared to patients without signs of life (45% versus 13%, p < 0.001), Fig. S2, B. A favorable 30-day survival was also seen in patients with a pre-hospital low-flow time < 60 min and in patients with a pre-hospital low-flow time > 80 min (40% versus 26%), Fig. S2, C. Patients with a prolonged pre-hospital low-flow time (> 80 min) had a higher rate of signs of life during CPR than patients with a pre-hospital low-flow time of 60–80 min (41% versus 31%, p = 0.14). For other subgroups, please refer to Additional files 2, 3: Figure S2 and Figure S3 for further 30-day survival data.

**Fig. 2:**
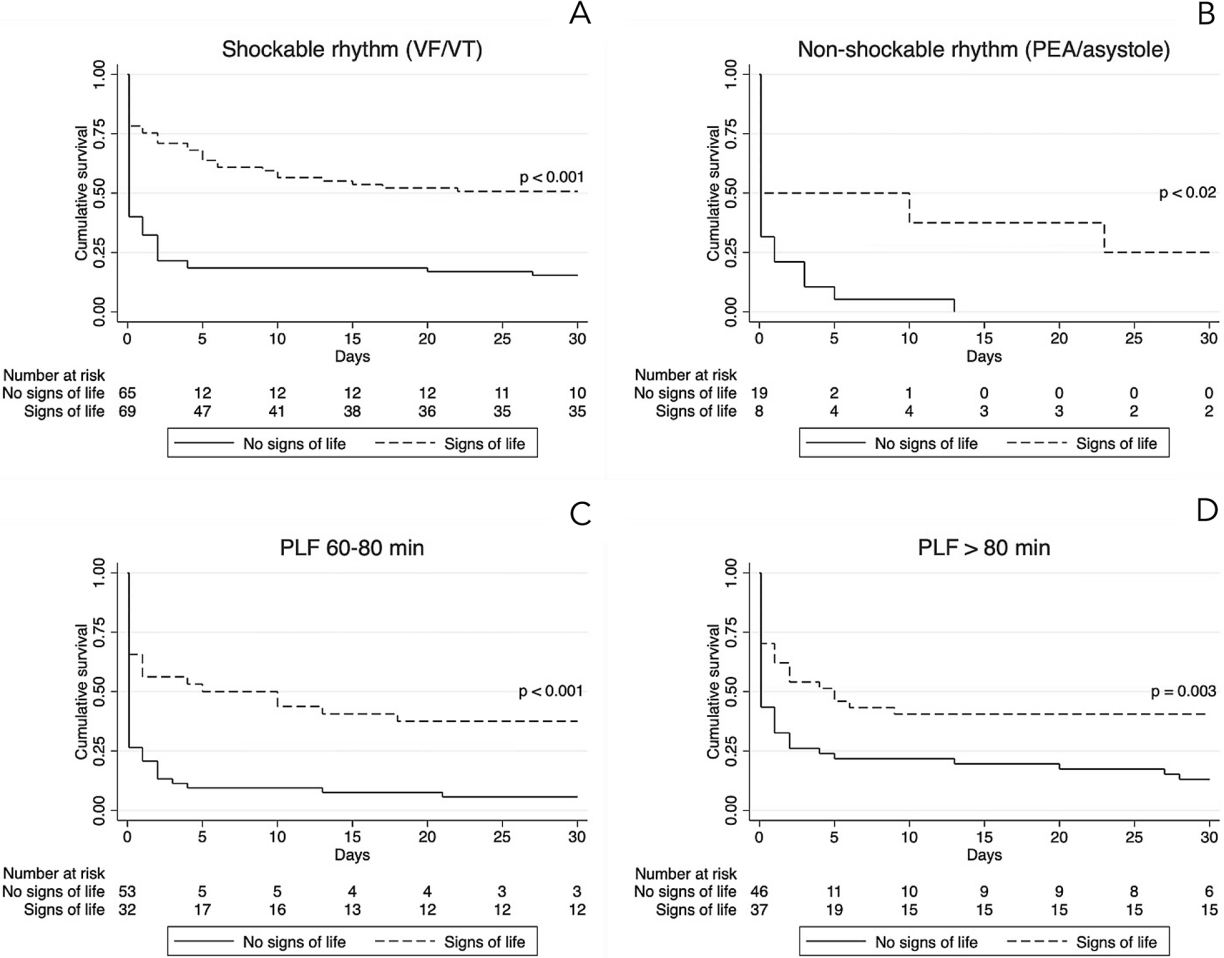
Kaplan–Meier survival curves for patients with and without signs of life during cardiopulmonary resuscitation with respect to initial presenting rhythm and pre-hospital low-flow times. **a** Patients with initial shockable rhythm with signs of life and no signs of life. **b** Patients with non-shockable rhythm with signs of life and no signs of life. **c** Patients with pre-hospital low flow (PLF) 60–80 min with signs of life and no signs of life. **d** Patients with PLF > 80 min with signs of life and no signs of life

Figure 2 demonstrates Kaplan–Meier curves for patients with and without signs of life during CPR and their 30-day survival with respect to initial presenting rhythm and pre-hospital low-flow times. Patients with signs of life had significantly higher survival rates compared to patients with no signs of life for both entities. Patients presenting non-shockable rhythm without signs of life had no survivors at day 30.

### Regional differences

Regional differences in patient selection and outcome are shown in Table 4. The ECPR activity per million inhabitants differed between centers. In most centers, younger age was a predominant factor for triage; nevertheless, 38 (15%) patients with age > 65 years did receive ECPR. Initial presenting rhythm differed significantly between centers (p = 0.003). Pre-hospital low-flow times were significantly longer (77 min versus 60 min, p = 0.02) in hospitals serving patients in remote and rural areas with a distance to center > 100 km (p = 0.001). Thirty-day survival between centers varied from 15 to 28%; however, this difference did not reach statistical significance, p = 0.41.

**Table 4:** Regional differences in triage and outcome of patients treated with extracorporeal cardiopulmonary resuscitation or Impella for refractory cardiac arrest

Variable	Aalborg University Hospital (n = 34)	Aarhus University Hospital (n = 138)	Odense University Hospital (n = 55)	Copenhagen University Hospital (n = 32)	*P-value*
No. of MCS/mio-inhabitants*year	11.6	11.2	8.9	2.8	< 0.001
Age < 65 years	26 (76)	113 (82)	51 (93)	31 (97)	0.03
Initial presenting rhythm					0.003
Shockable VT/VF	19 (56)	82 (59)	44 (80)	28 (88)	
PEA	11 (32)	38 (28)	4 (7)	4 (13)	
Asystole	3 (9)	18 (13)	7 (13)	0 (0)	
Unknown	1 (3)	0 (0)	0 (0)	0 (0)	
Witnessed arrest	31 (91)	114 (83)	49 (89)	29 (91)	0.38
Bystander CPR	31 (91)	134 (97)	52 (95)	29 (91)	0.42
No-flow ≥ 10 min	0 (0)	10 (7)	0 (0)	1 (3)	0.07
Pre-hospital low-flow (min)	60 [43–77]	75 [60–90]	77 [65–99]	60 [48–70]	< 0.001
Total low-flow (min)	90 [62–110]	105 [88–125]	119 [105–127]	94 [81–130]	< 0.001
Distance to center ≥ 100 km	2 (6)	21 (15)	19 (35)	0 (0)	0.001
30-day survival	5 (15)	38 (28)	14 (25)	9 (28)	0.41

Abbreviations: *MCS* Mechanical circulatory support*; VT* Ventricular tachycardia; *VF* Ventricular fibrillation; *PEA* Pulseless electrical activity*; CPR* Cardio pulmonary resuscitation

Values are stated as medians and interquartile range (IQR) or numbers and percentages. A p value of < 0.05 is considered significant

### Analysis of selection criteria in the Danish national consensus

**Fig. 3:**
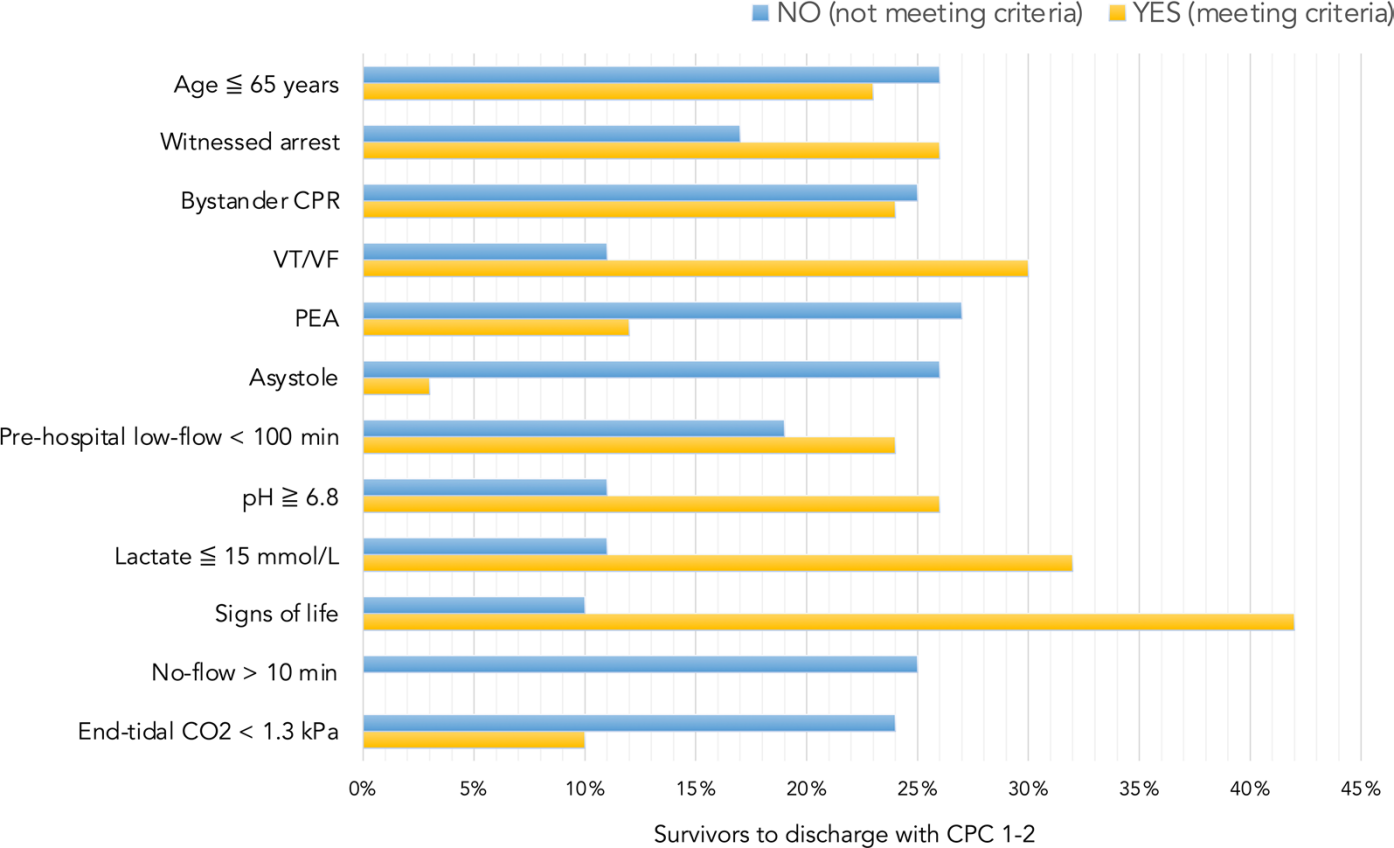
The impact of each selection criteria of the Danish national ECPR consensus in relation to survival to discharge with favorable neurological outcome (CPC 1–2). Survivors to discharge with CPC 1–2 in percentage when meeting selection criteria and failing to meet criteria of the national consensus

Figure 3 illustrates the survival rates of survivors to discharge with CPC 1–2 (n = 61) in regards to each selection criteria of the Danish 2018 national ECPR consensus. Poorest outcomes were seen in patients with initial non-shockable rhythm, no-flow times > 10 min and end-tidal CO_2_ < 1.3 kPa.

**Fig. 4:**
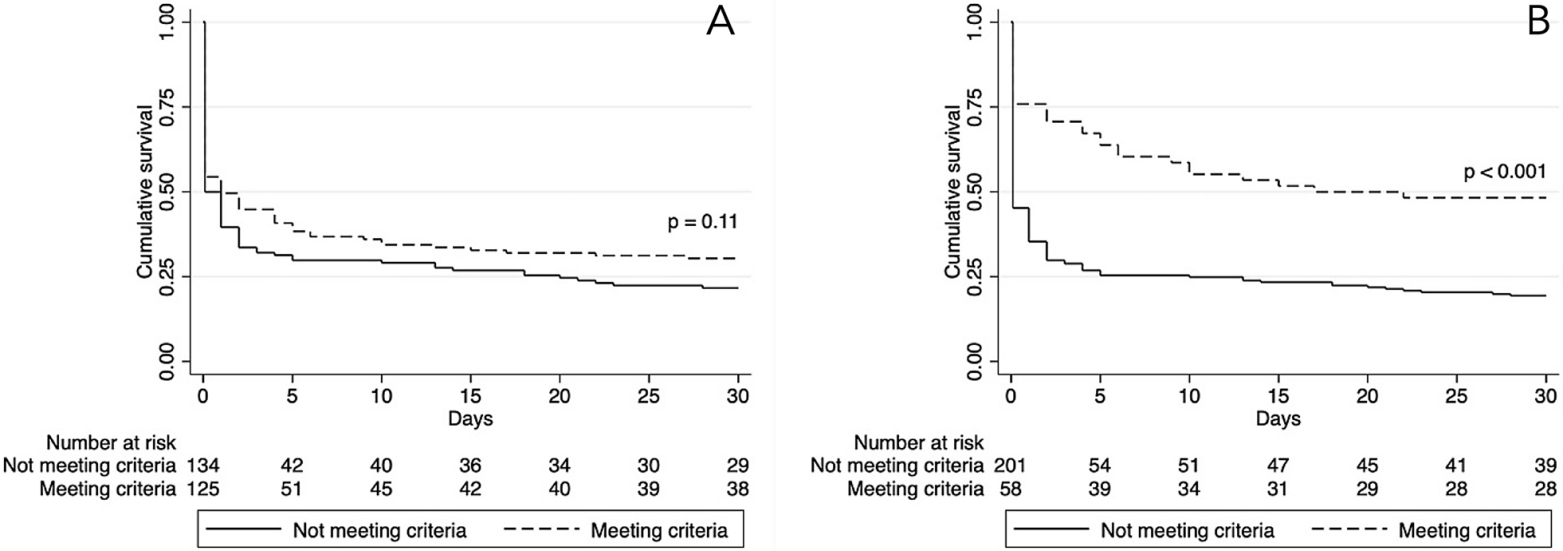
Kaplan–Meier survival curve for patients meeting the selection criteria and patients failing to meet the criteria. **a** Consensus A (National consensus): Survival analysis between patients meeting the Danish national consensus selection criteria in regards to younger age < 65 years, witnessed arrest, bystander CPR, initial shockable rhythm, no-flow < 10 min and end-tidal CO_2_ < 1.3 kPa, and patients failing to meet one or more criteria. **b** Consensus B (Extended version): Survival analysis based on a more refined assessment of selection criteria. Patients meeting all the selection criteria in the Danish national consensus and additionally one or more of following parameters: signs of life during CPR, pH > 6.8, lactate < 15 mmol/L and a pre-hospital low-flow < 100 min

Patients meeting all of the selection criteria of the Danish 2018 national ECPR consensus with respect to normothermic arrest of presumed cardiac origin, age < 65 years, witnessed arrest, bystander CPR, initial shockable rhythm, no-flow time < 10 min and end-tidal CO_2_ < 1.3 kPa (n = 125, 48%) were compared with those who failed to meet one or more of these parameters (n = 134, 52%) (Fig. 4). Thirty-day survival was 30% in patients who met the selection criteria compared with 22% in patients who failed to meet one or more criteria (p = 0.11).

A more refined assessment of selection criteria including parameters such as signs of life during CPR, pre-hospital low-flow < 100 min, initial pH > 6.8 and lactate ≤ 15 mmol/L in addition to the consensus criteria (Consensus B) showed significantly higher 30-day survival rates in patients meeting one or more of the extended criteria than in patients failing to comply with the criteria (48% versus 19%, p < 0.001) (Fig. 4). However, this also implied that 58% (39/67) of the 30-day survivors in this cohort would fail to meet the extended consensus.

## Discussion

The present study is the first nationwide multicenter study of outcomes in patients receiving MCS for refractory OHCA in Denmark. The main findings showed that one in four MCS patients survive to hospital discharge with a good neurological outcome. Outcome was significantly associated with initial presenting rhythm, signs of life during CPR, transient ROSC, pre-hospital low-flow time, initial pH and lactate levels. By offering MCS only to patients meeting the strictest criteria, survival exceeded 48%, but this occurred at the cost of withholding life-saving treatment in the majority of patients saved by MCS in the cohort. Thus, our data rightly questions the validity of stringent patient selection for MCS.

ECPR has emerged as a salvage therapy for patients suffering from refractory OHCA. Although resource demanding, ECPR has been shown to be both feasible and cost effective [13]. Moreover, recent small reports have described the potential of the Impella device as an alternative rescue therapy in cardiac arrest patients [7, 8]***.*** Several previous observational studies have demonstrated encouraging survival rates and a favorable neurological outcome from applying strict inclusion and exclusion criteria in distinctive patient populations [14–18]. The first randomized clinical trial recently published by Yannopoulos et al. (the ARREST trial) revealed superiority of ECMO-facilitated resuscitation with a survival rate of 43% compared with 7% with standard advanced life support [6]. Survival at six months was also greater in the ECMO group (hazard ratio (HR) 0.16, 95% CI 0.06–0.41, p = 0.0001). Whereas the trial was well-designed and supports use of ECPR in refractory OHCA, apparent limitations are present as this was an open-labelled single-center study with a relatively small and highly selected patient population.

In Denmark, a number of initiatives to improve pre-hospital quality of care for OHCA patients have produced a remarkable increase in 30-day survival rates from 3.9% in 2001 to 16% in 2018 [19]. Concurrently, MCS with ECMO and in limited cases the Impella device, has been evolving steadily and is now an established treatment for selected patients with refractory OHCA in all Danish regions. However, given the formal inclusion and exclusion criteria defined in the Danish national ECPR consensus, one might assume that the results of our study would reflect a more homogeneous population. Despite our intention to select qualified candidates, violation of the national consensus was still seen in 52%. In this population, the fatal consequence of treatment not being provided, opted some ECPR teams to perform MCS despite adverse conditions. Selection of appropriate candidates for MCS is evidently a challenge in real clinical practise. Despite growing interest in and a growing body of literature on MCS for refractory OHCA, robust evidence on patient eligibility is still lacking.

In the present study, the majority of patients treated with MCS were younger (< 65 years) in compliance with the national consensus. This may explain why no statistically significant difference was detected in age between survivors and non-survivors. One may have predicted a worse prognosis in patients above 65 years of age, but survival to hospital discharge with good neurological outcome was similar in this group compared with the younger population < 65 years (26% versus 23%). Previous studies have proposed advanced age as a predictor of a poor outcome in patients with ECPR [20], and some studies have even suggested an age of > 75 years as a contraindication for ECPR [21], which is in line with our results. In our cohort, no one above 72 years of age survived to discharge. However, there is limited and conflicting evidence that older age is associated with poor outcome [22, 23]. The dilemma of initiating ECPR in the elderly remains controversial and more evidence is needed to confirm, whether to proceed or stop advanced life support based on age limits.

Pre-hospital parameters are crucial in the selection of suitable candidates for ECPR. Initial shockable rhythm, transient ROSC and signs of life during CPR are considered favorable prognostic factors in ECPR [24–26]. In a prospective registry study, Bougouin et al. compared conventional CPR with ECPR in 13,191 consecutive patients with OHCA [27]. Prognostic factors in the ECPR group comprised an initial shockable rhythm (OR 3.9, 95% CI 1.5–10.3) and transient ROSC (OR 2.3, 95% CI 1.1–4.7) prior to ECPR implementation. One of the main findings in our study was that patients with signs of life during CPR had a threefold higher survival rate than patients without signs of life during CPR. This is in correlation with a study recently published by Debaty et al. [28] The authors found that any signs of life before or during CPR substantially improved 30-day survival with favorable neurological outcome in a multivariable prognostic model (OR 7.35, 95% CI 2.71–19.97). These results are supported by our data. Signs of life was a highly significant prognostic factor in relation to initial presenting rhythm and low-flow times. In the present cohort, a pre-hospital low-flow time < 60 min was associated with an increased survival rate; however, patients exceeding pre-hospital low-flow times > 80 min also had an advantageous outcome. Patients with pre-hospital low-flow times > 80 min had a higher rate of signs of life during CPR than patients with a pre-hospital low-flow time of 60–80 min. The presence of signs of life increased survival rates substantially in both groups. Collectively, these observations support the evidence of incorporating signs of life as an important factor in the selection of patients for ECPR. Our results suggest that prolonged resuscitation efforts in the field may not be futile, especially in patients presenting favorable circumstances such as signs of life and where ECPR can be established within a reasonable timeframe. The present findings do not allow us to determine whether any of the patients would have survived without MCS. However, the long total low-flow times observed makes this unlikely.

Historically, the arrest-to-perfusion time has been linked to survival [16, 18, 29, 30]. Wengenmayer et al. reported that among 133 patients with cardiac arrest treated with ECPR, low-flow time was an independent predictor of mortality [30]. Bartos et al. demonstrated a significant association between time from arrest to sufficient ECPR flow and neurological outcome in a cohort of 160 patients [18]. These results are similar to ours. In the present study, hospitals serving patients in remote and rural areas had longer arrest-to-perfusion time due to longer distances to the invasive center. Implementation of systematic pre-hospital ECPR calls, more rapid allocation of helicopter-mediated transport and direct triage to the catheterization laboratory may improve the performance and facilitate a reduction in system delay for these patients. In this setting, pre-hospital ECPR may also shorten the interval from collapse to onset of ECPR [17].

The predictive value of pH and lactate levels in patients with cardiac arrest is well established. Controversy still exists regarding the ECPR population. In our study, initial arterial pH and lactate levels were found to be associated with mortality. This finding is consistent with previous findings [14, 31]. Jung et al. retrospectively reviewed 93 patients with cardiac arrest undergoing ECPR and found results similar to our results [32]. On the contrary, Leick et al. found no association between elevated lactate levels and mortality [33]. Our results support the inclusion of pH and lactate into our decision-making when considering patients for MCS, whereas specific cutoffs still need conformation in other cohorts. Importantly, a stringent use of pH > 6.8 and lactate < 15 mmol/L as selection criteria, may result in denying life-saving therapy to a considerable number of the survivors present in this cohort.

In the present study, we assessed pre-hospital and in-hospital factors in relation to outcome, which may come in benefit for clinicians in the selection of appropriate candidates for MCS. High mortality rates were seen among patients with initial non-shockable rhythm, no-flow times > 10 min and end-tidal CO_2_ < 1.3 kPa. On the contrary, our results suggest that a more refined assessment of the inclusion criteria, comprising additional criteria such as signs of life during CPR and lactate levels, may improve survival rates in patients receiving MCS. Nevertheless, one must recognize that limiting patient selection to strict pre-defined criteria will inevitably exclude some patients in whom MCS would have bought valuable time until the reversible cause could be treated. The fact that the national consensus was violated in 52% of patients, of whom 20% survived to hospital discharge with a good neurological outcome in 94% of the cases, indicates that there must be room for individual decision-making, especially in the young patients. Patient selection for MCS continues to be a challenging part of real-world clinical practice and further randomized clinical trials are warranted.

## Limitations

The present study has several limitations. Its retrospective nature makes it subject to patient selection bias. The national consensus was available and adopted to some extent in all centers. This produces risk of bias in the evaluation of the associations with outcome. Although we conducted a multicenter study using nationwide registry data, the heterogeneity of the study population with a mixed cohort of patients with OHCA hampers generalization of the results. Neurological outcome at hospital discharge is a fairly crude measure; it is, however, broadly used in cardiac arrest studies. Studies assessing long-term survival and neurological outcome are necessary.

## Conclusion

Patients receiving MCS for refractory OHCA presented promising survival rates with a favorable neurological outcome at hospital discharge. Even though a more stringent patient selection with additional criteria may produce higher survival rates, this would also limit the number of candidates and possibly exclude half of the survivors from treatment, why optimization of the selection criteria is still of essence in the future.

## Supplementary Information

**Additional file 1. Table S1**: National and regional indications for mechanical circulatory support in refractory normothermic cardiac arrest with presumed cardiac origin.

**Additional file 2. Figure S1**: National trend in the use of mechanical circulatory support for OHCA in Denmark.

**Additional file 3. Figure S2**: Kaplan-Meier survival curves of patients who had out-of-hospital cardiac arrest and received mechanical circulatory support.

**Additional file 4. Figure S3**: Kaplan-Meier survival curves stratified by groups.

## Data Availability

The data underlying this article were provided by the administrative Regions of Denmark under license from the Danish Data Protection Agency and the Danish Patient Safety Authority and cannot be shared publicly due to Danish regulations for data protection. Data are however available from the authors upon reasonable request and with permission from the five administrative Regions of Denmark.

## Abbreviations

CPC: Cerebral performance category
CPR: Cardiopulmonary resuscitation
ECMO: Extracorporeal membrane oxygenation
ECPR: Extracorporeal cardiopulmonary resuscitation
ICU: Intensive care unit
LUCAS: Lund university cardiopulmonary assist system
MCS: Mechanical circulatory support
OHCA: Out-of-hospital cardiac arrest
PEA: Pulseless electrical activity
ROSC: Return of spontaneous circulation
TTM: Targeted temperature management
VF: Ventricular fibrillation
VT: Ventricular tachycardia
