# Supplementary material for: Mechanical circulatory support for refractory out-of-hospital cardiac arrest: a Danish nationwide multicenter study
Source: Crit Care. 2021 May 22;25:174. doi: 10.1186/s13054-021-03606-5 (PMC8141159; doi:10.1186/s13054-021-03606-5)
Supplement: Supplementary file 1 — Additional file 1. Table S1: National and regional indications for mechanical circulatory support in refractory normothermic cardiac arrest with presumed cardiac origin. [file 13054_2021_3606_MOESM1_ESM.docx]

**TABLE S1** National and regional indications for mechanical circulatory support in refractory normothermic cardiac arrest with presumed cardiac origin

| Parameter | Aalborg University Hospital | Aarhus University  Hospital | Odense University Hospital | Copenhagen University Hospital |
| --- | --- | --- | --- | --- |
| NATIONAL INDICATIONS |  |  |  |  |
| Age < 65 years | + | + | + | + |
| No or few comorbidities | + | + | + | + |
| Initial shockable rhythm  (in rare cases PEA) | + | + | + | + |
| Witnessed arrest | + | + | + | + |
| Bystander CPR | + | + | + | + |
| ET CO_2_ > 1.3 kPa | + | + | + | + |
| REGIONAL VARIANCES |  |  |  |  |
| Pre-hospital low-flow < 100 min | - | - | + | + |
| pH > 6.8 | + | + | - | - |
| Lactate < 15 mmol/L | + | + | - | - |
| Signs of life during CPR | + | + | + | - |

**PEA** Pulseless electrical activity; **CPR** Cardiopulmonary resuscitation
