# Supplementary material for: Mechanical circulatory support for refractory out-of-hospital cardiac arrest: a Danish nationwide multicenter study
Source: Crit Care. 2021 May 22;25:174. doi: 10.1186/s13054-021-03606-5 (PMC8141159; doi:10.1186/s13054-021-03606-5)
Supplement: Supplementary file 2 — Additional file 2. Figure S1: National trend in the use of mechanical circulatory support for OHCA in Denmark. [file 13054_2021_3606_MOESM2_ESM.docx]

**Additional file 2 (Supplementary)**

**FIGURE S1.** National trend in the use of mechanical circulatory support for OHCA in Denmark


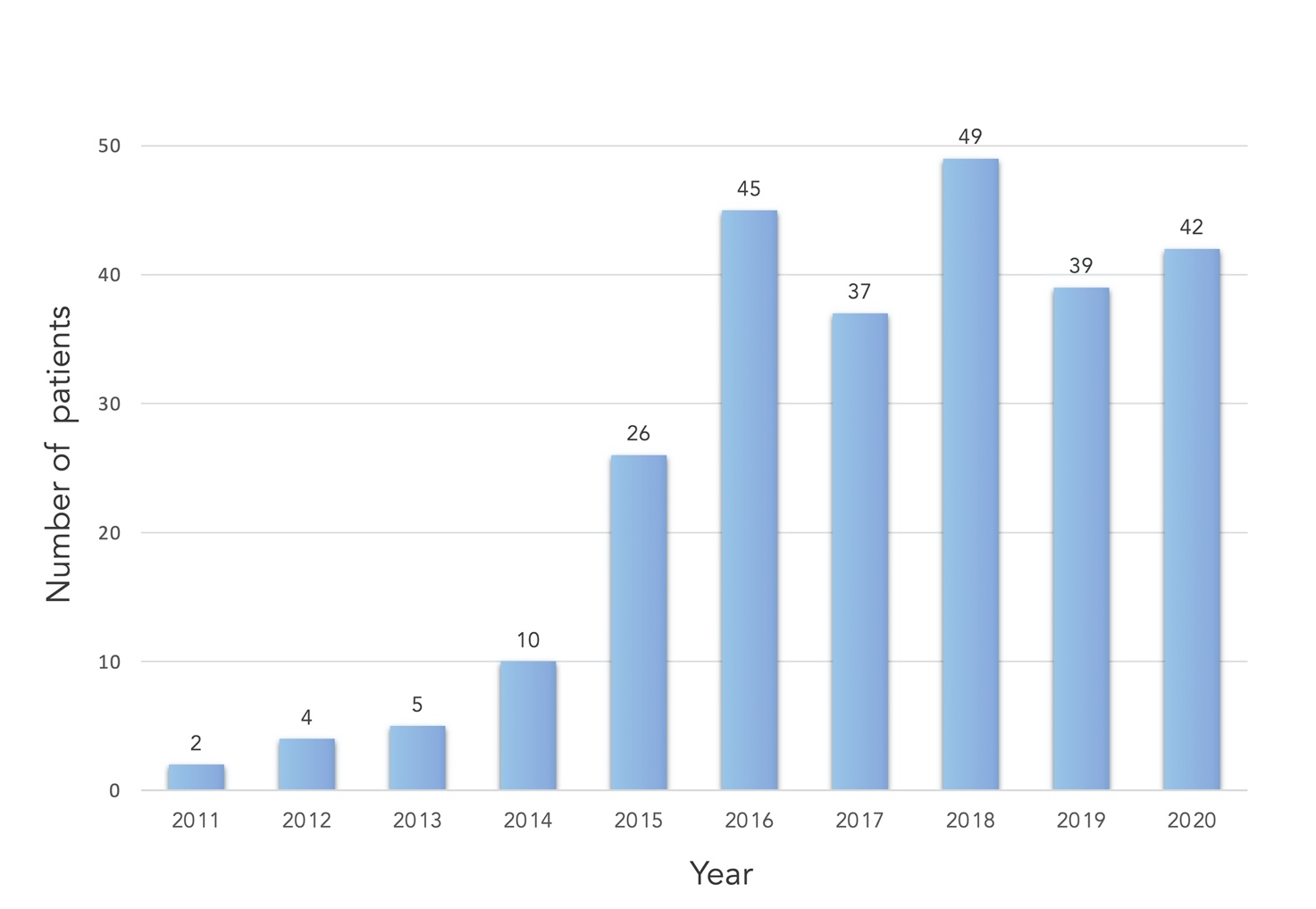


The temporal use of mechanical circulatory support in four cardiac centres in Denmark from July 2011 to December 2020.
