# Supplementary material for: Mechanical circulatory support for refractory out-of-hospital cardiac arrest: a Danish nationwide multicenter study
Source: Crit Care. 2021 May 22;25:174. doi: 10.1186/s13054-021-03606-5 (PMC8141159; doi:10.1186/s13054-021-03606-5)
Supplement: Supplementary file 3 — Additional file 3. Figure S2: Kaplan-Meier survival curves of patients who had out-of-hospital cardiac arrest and received mechanical circulatory support. [file 13054_2021_3606_MOESM3_ESM.docx]

**Additional file 3 (Supplementary)**

**FIGURE S2.** Kaplan-Meier survival curves of patients who had out-of-hospital cardiac arrest and received mechanical circulatory support


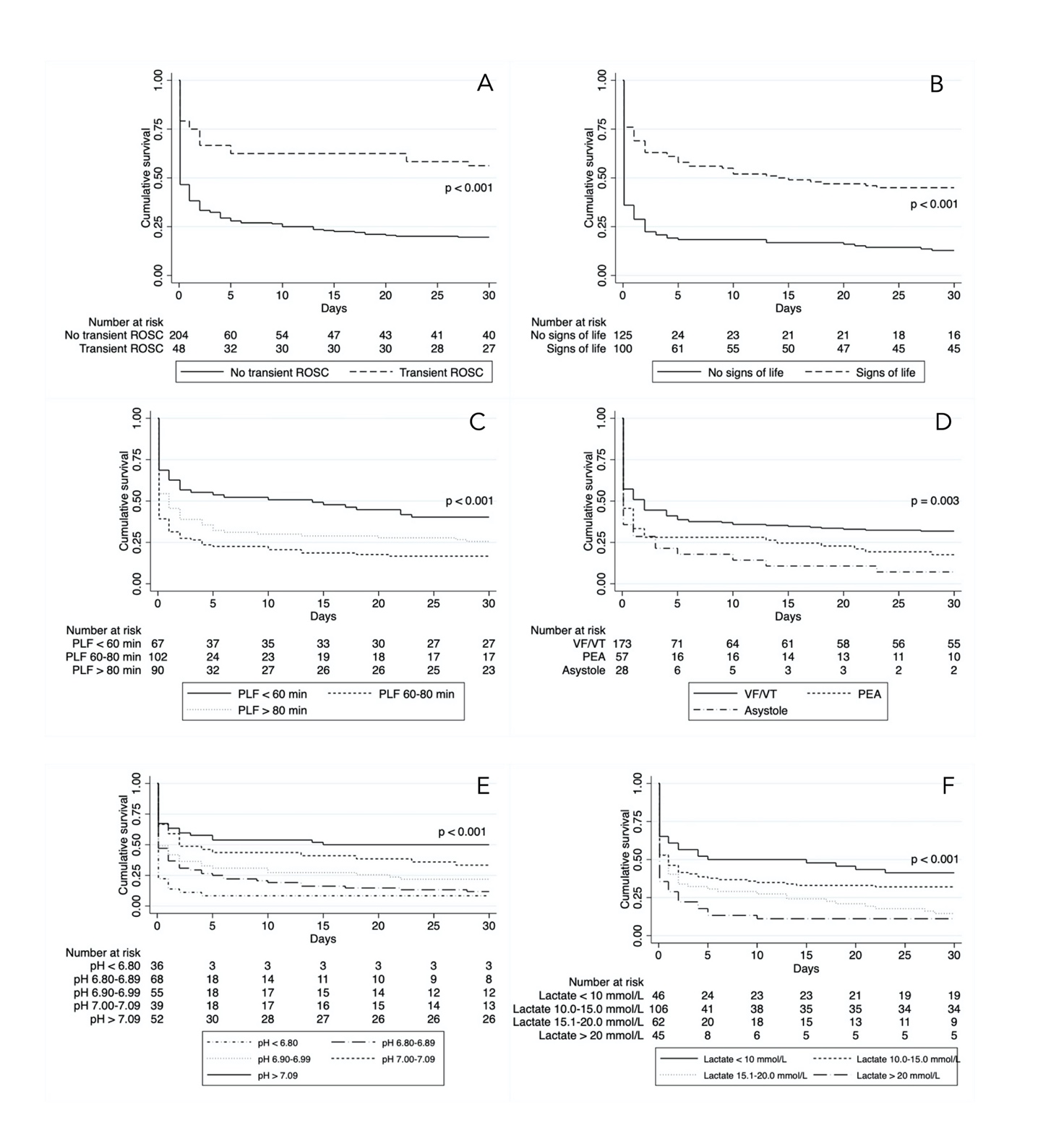


(A) Patients with transient ROSC versus patients with no ROSC prior to mechanical circulatory support. (B) Patients with signs of life during cardiopulmonary CPR versus no signs of life during CPR prior to mechanical circulatory support. (C) Patients stratified by pre-hospital low-flow time. (D) Patients stratified by initial presenting rhythm. (E) Patients stratified by initial pH levels prior to mechanical circulatory support. (F) Patients stratified by initial lactate levels prior to mechanical circulatory support.

**ROSC** Return of spontaneous circulation**; CPR** Cardiopulmonary resuscitation; **PLF** Pre-hospital low-flow; **VT** Ventricular tachycardia; **VF** Ventricular fibrillation**; PEA** Pulseless electrical activity
