# Supplementary material for: Mechanical circulatory support for refractory out-of-hospital cardiac arrest: a Danish nationwide multicenter study
Source: Crit Care. 2021 May 22;25:174. doi: 10.1186/s13054-021-03606-5 (PMC8141159; doi:10.1186/s13054-021-03606-5)
Supplement: Supplementary file 4 — Additional file 4. Figure S3: Kaplan-Meier survival curves stratified by groups. [file 13054_2021_3606_MOESM4_ESM.docx]

**Additional file 4 (Supplementary)**

**FIGURE S3** Kaplan-Meier survival curves stratified by groups

**
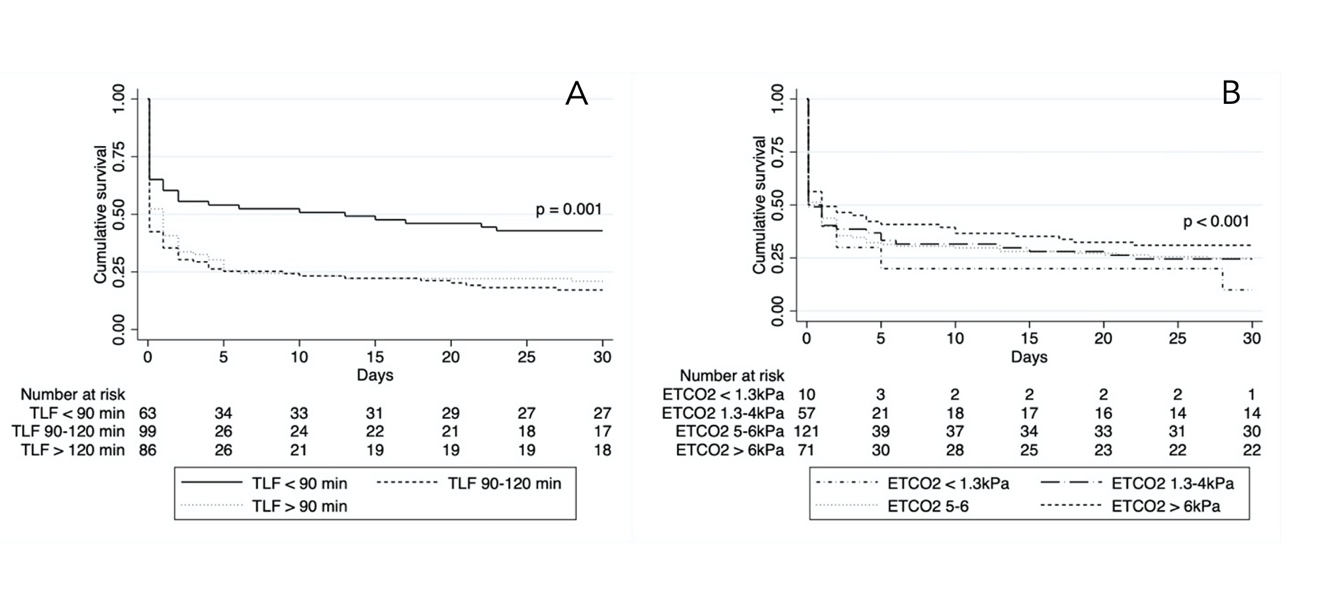
**

1. Patients stratified by total low-flow time (TLF), (B) Patients stratified by end-tidal CO2 (ETCO2).
